# Sensor Fusion and Convolutional Neural Networks for Indoor Occupancy Prediction Using Multiple Low-Cost Low-Resolution Heat Sensor Data

**DOI:** 10.3390/s21041036

**Published:** 2021-02-03

**Authors:** Simon Arvidsson, Marcus Gullstrand, Beril Sirmacek, Maria Riveiro

**Affiliations:** Jönköping AI Lab (JAIL), Department of Computer Science and Informatics, School of Engineering, Jönköping University, 551 11 Jönköping, Sweden; arsi1632@student.ju.se (S.A.); guma1502@student.ju.se (M.G.); maria.riveiro@ju.se (M.R.)

**Keywords:** heat sensors, multi-sensor, sensor fusion, occupancy prediction, machine learning, artificial intelligence (AI), neural networks, smart offices

## Abstract

Indoor occupancy prediction is a prerequisite for the management of energy consumption, security, health, and other systems in smart buildings. Previous studies have shown that buildings that automatize their heating, lighting, air conditioning, and ventilation systems through considering the occupancy and activity information might reduce energy consumption by more than 50%. However, it is difficult to use high-resolution sensors and cameras for occupancy prediction due to privacy concerns. In this paper, we propose a novel solution for predicting occupancy using multiple low-cost and low-resolution heat sensors. We suggest two different methods for fusing and processing the data captured from multiple heat sensors and we use a Convolutional Neural Network for predicting occupancy. We conduct experiments to assess both the performance of the proposed solutions and analyze the impact of sensor field view overlaps on the prediction results. In summary, our experimental results show that the implemented solutions show high occupancy prediction accuracy and real-time processing capabilities.

## 1. Introduction

Predicting occupancy plays a crucial role in managing smart buildings security, energy consumption, efficient use of the facilities, and the optimization of the automation techniques. For many years, occupancy prediction models have been used to automate heating, ventilation, and air conditioning (HVAC) in building management systems in order to reduce energy consumption. Buildings use approximately 40% of the global energy and they are responsible for almost one-third of the worldwide greenhouse gas emissions, utilizing about 60% of the world’s electricity. It is shown that more than 50% of the energy that is consumed in a building could be saved if occupants and their activities were considered in the automatic processes that are related to energy consumption [1]. Indoor occupancy prediction is also very relevant in the current societal challenges that are associated with the Covid19 outbreak where controlling, for example, human presence and activities in non-residential buildings can prevent dense crowds.

Ambient intelligence is commonly used when referring to ambient sensor vision-based systems that naturally blend with our everyday life [2]. One of the most important qualities of the ambient sensors is that they do not enable personal identification. Therefore, they can be used in non-residential buildings without interfering with any privacy regulations. Moreover, ambient sensors are much cheaper than high-resolution cameras, which makes it possible to distribute multiple sensors in buildings that cover large areas without dramatically increasing the costs.

However, analyzing the data that are generated by these low-cost and low-resolution ambient sensors for covering large areas is not exempt from challenges. For example, there are many challenges associated with how to fuse or integrate the data from all of these low-resolution sensors. In this paper, we propose an Artificial Intelligence-based and, even more specifically, a Neural Network-based (NN-based) method for predicting occupancy from low-cost low-resolution heat sensor data. We present and describe two different workflows for fusing multiple sensor data in order to be able to cover larger areas in our predictions. In particular, this paper provides the following contributions:

- We propose two different sensor fusion methods to combine the multi-sensor information, and we compare these two methods by discussing their advantages and disadvantages for various environments and situations.
- We propose a novel NN-based occupancy prediction method for indoor occupancy prediction while using the combined multi-sensor information.
- We discuss the advantages and disadvantages of using a NN-based occupancy prediction method; instead, the earlier presented [3] machine learning (ML)-based and computer vision (CV)-based solutions for occupancy prediction in large spaces using multiple sensors.
- We discuss the implications of the different sensor fusion and sensor positioning results for a potential algorithm design to solve the optimal sensor placement problem.

This paper is organized, as follows. Section 2 presents the relevant information regarding occupancy prediction and multi-sensor fusion techniques for extending the prediction models to large areas. We pay closer attention to methods that employ heat sensors in Section 2.2, while we address earlier studies on low-resolution data processing methods using AI-techniques in Section 2.3. Section 3 outlines the objectives and research questions of our experimental study. In Section 4, we describe the experimental environment, the sensor placements, and the data collection scenarios. We introduce our multi-sensor data fusion workflows and our NN-based occupancy prediction method step by step in Section 5. We present the quantitative results of our experiments shown in Section 6. The results obtained are discussed in light of relevant literature in Section 7. Finally, the limitations and future work are presented in Section 8/ Section 9 concludes this paper with some final remarks.

## 2. Background

Indoor occupancy prediction is an active and open research topic, with multiple solutions being described in the literature. Besides particular methods, several overviews and literature studies have been published, covering the most frequent methods, advantages and disadvantages of each method, and research challenges in the field. This section provides a short summary of these studies.

### 2.1. Occupancy Prediction in Indoor Environments

Most of the papers that provide solutions to the problem of indoor occupancy prediction, for instance [4,5], provide a comparison of different ambient sensors and the data processing methods. Table 1 lists and describes the most commonly used ambient sensors, while also providing information regarding their area coverage differences.

Passive infrared (PIR) sensors can be used to detect the presence and absence of occupants in an office. Raykov et al. [6] used a single PIR sensor for occupancy estimation inside a room. They trained a machine learning (ML) algorithm to learn motion patterns, and then used the model as a feature to predict the number of occupants. However, it is unclear whether this method generalizes well to other environments or to larger office spaces.

A smart meter is an electronic device that records information, such as the consumption of electric energy, voltage levels, current, and power factor. It can only detect the presence or absence of people within an area, rather than providing information regarding the number of people. Fan et al. [7] proposed methods for analyzing smart meter data and they have even used forecasting techniques to estimate the future energy consumption. Razavi et al. [8] proposed a smart meter-based occupancy prediction method to estimate the number of occupants in a large building. The major challenge of the smart meter-based solution is that each building might have different energy needs, depending on the activities of the occupants. For instance, if the occupants of a building are computer programmers, each of them would need a computer and other electronic facilities around. However, a building that is an art studio where the occupants do paintings, acting, or dancing, their electricity needs would be totally different. Therefore, the main challenges of these solutions are the calibration of the smart meters and generalization of the developed models.

CO${}_{2}$ sensors can be used in order to also obtain a rough estimation of occupancy. Ang, Salim, and Hamilton [9] have proved that CO${}_{2}$ rate, illumination level and sound rate are the top three most dominant features to detect indoor human occupancy, with the CO${}_{2}$ rate being the most dominant one. This work presents very interesting results, showing an almost linear correlation between the number of people in a room and the CO${}_{2}$ rate. However, the experiments were conducted in one room and larger area coverage was left as future work. Moreover, the authors have expressed their concerns regarding the general applicability of their proposed algorithm, for instance, to larger or smaller rooms.

Heat sensors provide images as the standard cameras do. However, ambient low-cost heat sensors that are used for occupancy prediction provide very low-resolution images (in our case, 8×8 pixels). These images show the heat of the objects that are within the field of view. Because of the body heat, humans appear in these images like two or three highlighted pixels, which definitely is not enough for identification. In our earlier study [3], we provided extensive background research in the field of occupancy prediction while using such heat sensors. In addition to that, we have proposed two novel occupancy prediction methods using a low-resolution heat sensor. The first method was based on the usage of traditional computer vision techniques, while the second one was based on a machine learning based classification method that was trained with the features that we have extracted from the heat sensor images. We have compared these two methods to discuss their advantages and disadvantages in detail. Even though both of the methods provided reliable results showed through our experiments conducted in a smart office, we could not extend the algorithms to make occupancy prediction in larger areas.

An extensive literature review on occupancy prediction methods for non-residential buildings comparing their advantages and disadvantages is presented in [10]. The authors also offered their occupancy prediction solution employing high resolution cameras. Because the authors were aware of the privacy and security issues of their solution, they proposed an encryption method to store the occupancy prediction data by blocking the pixels where people were observed. Mulia et al. [11] presented another extended review on building occupancy methods and the frequently used sensors. They grouped existing occupancy prediction methods in statistical and machine learning-based models. Their review showed that, as compared to other ambient sensors, heat sensors are used in a very limited number of applications. This is probably because other sensors (like CO${}_{2}$, sound, light) can provide information regarding larger areas (like the whole room), however, a heat sensor can only observe the occupancy in a small section of a room. When monitoring large areas with heat sensors, the challenges of sensor fusion need to be addressed.

Chen et al. [4] provide an additional review on occupancy estimation for smart buildings. Because different sensors have different strengths and weakness, they provide a categorization of occupancy prediction methods based on different types of sensors. Similarly, Saha et al. [5] present an overview of methods for occupancy prediction, but also review those that are based on person counting and tracking within large buildings. They focused on mathematical methods to explain different approaches in order to solve the problem (including data collection, preparation and cleaning steps which were not compared earlier) and explain the different quantitative analysis metrics.

Particular solutions for occupancy prediction using NNs are the works by Jiang et al. [12] and Zuraimi et al. [13]. Jiang et al. [12] propose a NN-based solution to predict occupancy from CO${}_{2}$ sensors and show that it is possible to reach good accuracy values. Zuraimi et al. [13] use a similar NN-based solution in a larger auditorium that was visited by a large number of people. They discuss the effects of ventilation and whether the high amount of people within the room make a linear impact on the CO${}_{2}$ level or not. Finally, they elaborate on the reliability of using CO${}_{2}$ sensors in such large public areas where crowds are gathered.

### 2.2. Multi-Sensor Fusion for Large Indoor Areas

How to fuse and integrate the data that are generated by multiple sensors is a key challenge when predicting occupancy in large monitored areas. Sensor fusion is described as “the combining of sensory data or data derived from sensory data such that the resulting information is in some sense better than would be possible when these sources were used individually” [14]. Durrant-Whyte [15] summarized the sensor fusion algorithms under three different categories as complementary fusion, competitive fusion, and cooperative fusion. A sensor configuration is called complementary if the sensors do not directly depend on each other, but can be combined in order to give a more complete image of the phenomenon under observation. This resolves the incompleteness of the sensor data. The employment of multiple cameras, each observing disjunct parts of a room, is an example of a complementary configuration. Generally, fusing complementary data is easy, since the data from independent sensors can be appended to each other. Sensors in a competitive configuration have each sensor delivering independent measurements of the same property. Competitive configurations are used for fault-tolerant and robust systems. The reduction of noise by combining two overlaying camera images is an example of such a configuration. A cooperative sensor network uses the information that is provided by two independent sensors to derive information that would not be available from the single sensors. An example of a cooperative sensor configuration is stereoscopic vision—by combining two-dimensional images from two cameras at slightly different viewpoints, a three-dimensional image of the observed scene can be derived.

In the field of smart offices and buildings, most of the research studies for occupancy prediction have used multi-sensor fusion methods, not for fusing the same type of sensors, but for fusing different types. The reason is that ambient sensors provide limited reliability with their low-resolution, small field of view, and less reliable information, which might be affected by environmental changes. Each sensor has some unique properties and limitations for occupancy estimation and detection. The fusion of multiple sensor types can boost the performance of occupancy estimation and detection by taking advantages and compensating the limitations of each sensor.

Many advanced sensor fusion algorithms have been proposed in the literature for the problem of occupancy prediction. We believe that most of these solutions fall under the competitive sensor fusion category. For instance, Candanedo and Feldheim [16] proposed an occupancy detection system while using environmental CO${}_{2}$ sensors, light, temperature, and humidity. Mao et al. [17] fused information coming from CO${}_{2}$ and light sensors in order to predict occupancy of a large building. They used the prediction results for automation of the heating, ventilation, and illumination facilities of the building rooms. Abade et al. [18] used multiple and different type of ambient sensors to develop a machine learning-based solution to predict occupancy; they employed outdoor and indoor thermal sensors, as well as indoor CO${}_{2}$ sensors. The indoor sensors were randomly distributed; therefore, sensor distribution was not discussed in their work. The synchronized multi-sensor data from both indoor and outdoors were used for training a classifier that can estimate the indoor occupancy. Yang et al. [19] used similar multi-sensor data input of a larger indoor environment and trained a NN (Radial Basis Function, RBF). Wang et al. [20] used a similar multi-sensor system for estimating the cooling demand of indoor environments. A relevant interesting review in this regard is the work presented by Ahmad et al. [10], which summarizes the most commonly used ambient indoor sensors in multi-sensor occupancy prediction applications. The authors concluded that there is no single method or sensor that is identified as the best way to estimate indoor occupancy. Therefore, there is still a need for simplified, secure, energy efficient, and highly accurate methods for multi-sensor occupancy prediction.

To the best of our knowledge, multi-sensor based occupancy prediction in large areas only using heat sensors (and not other sensor types) has not been addressed in previous studies. While most of the previously proposed algorithms fall in the competing fusion category, we believe that our study falls in both the complementary and cooperative fusion categories: complementary fusion, since one sensor sees the areas that are not seen by other sensors and cooperative fusion, since the overlapping sensor views are used for improving the image qualities in those areas (not relying on one sensor more than another like competing fusion methods do). We introduce the details of our suggested solutions in Section 5.

### 2.3. Using AI Techniques for Occupancy Prediction from Low-Resolution Sensors

In the last decade, AI-based models, and especially convolutional NNs (CNNs), have been frequently used in order to solve computer vision problems because of their capabilities of learning the best parameters of the optimal solution to a good generalization. However, in most of the studies, these models are trained with high-resolution images that hold features that can represent fine features (like corners, edges, and texture) of the objects within. Cai et al. [21] used a CNN model to classify very low-resolution (9×9 pixel size) camera images. Even though their input resolutions were close to our heat sensor images, RGB bands of the normal camera image still provided sharper details about the objects within the images. Chevalier et al. [22] used remote sensing images as low-resolution image examples, since the objects, like airplane and cars, are only represented with few pixels within these images. Even though their images were grey scale (i.e., no RGB bands were available), they still contained sharper object features than heat camera images which vaguely represent object borders. Chevalier et al. [22] compared a classifier that was trained with images having less than 50×50 pixel resolutions with another classifier that was trained with images having higher than 100×100 pixel resolutions. They showed that the performance decreased when the input image resolutions become smaller. To the best of our knowledge, we have not seen examples of NN based classifiers that were trained with 8×8 pixel size heat sensor images. Therefore, in this paper, we tackle the challenge of investigating potential NN-based solutions for predicting occupancy while using low-resolution sensor data.

## 3. Aim and Objectives

Even though several solutions exist for occupancy prediction, there are several challenges that are still unresolved, for instance, the performance is still far from satisfactory for many applications [4], the images used from heat sensors are normally of high resolution, and the sensor fusion solutions are typically of competing fusion type, as we showed in the previous section.

Thus, in this study, we aim at achieving three overall goals:

- To propose a new NN model to predict occupancy in real-time using fused low-resolution heat sensor data.
- To use multiple heat sensors and suggest new fusion techniques (both collaborative and cooperative) in order to predict occupancy in large areas.
- To analyze and understand the impact that the sensor field of view overlap has on occupancy prediction accuracy.

In the following sections, we introduce the data sets, the proposed solutions, and the experiments that were conducted in order to answer these questions.

## 4. Scenario and Data

**Figure 1:**
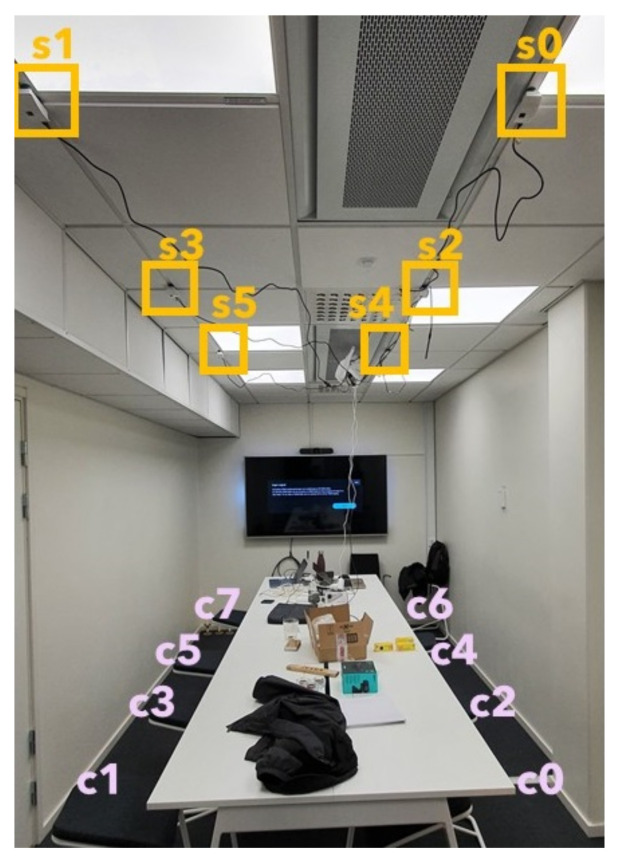
Setup of the test room. The numbered multiple heat sensor are seen on the ceiling. The chairs are numbered in order to keep the occupant positions in the data set for validation.

**Figure 2:**
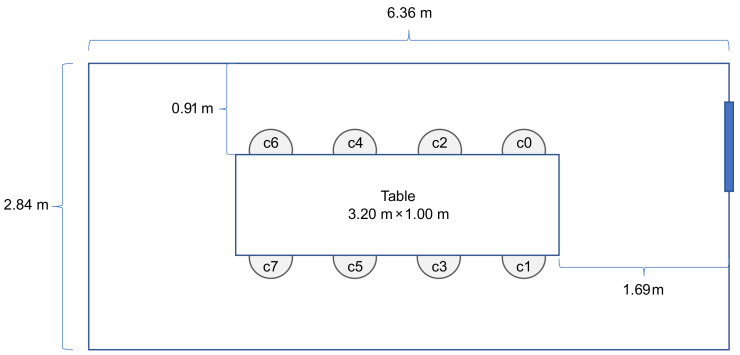
Floorplan of the room used in the study showing seats c0–c7.

**Figure 3:**
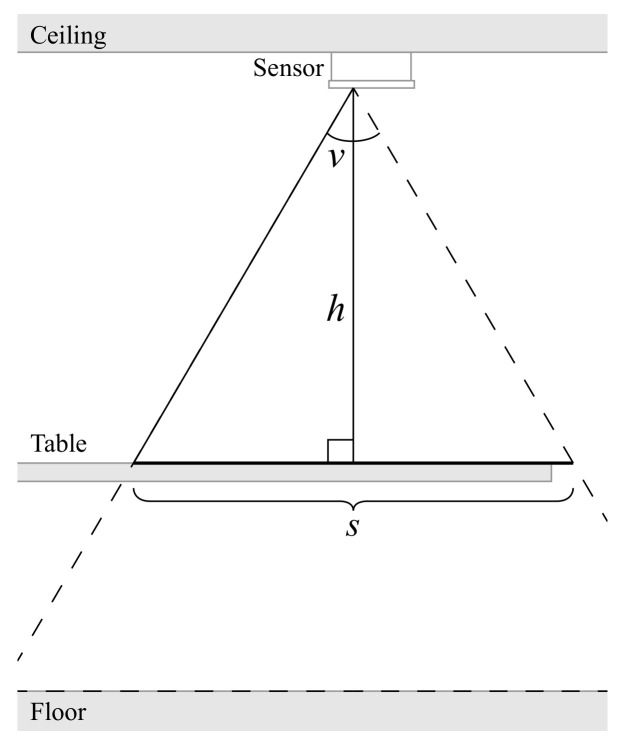
Side view of the room, showing the relationship between field of view, height to the table, and viewing area.

**Figure 4:**
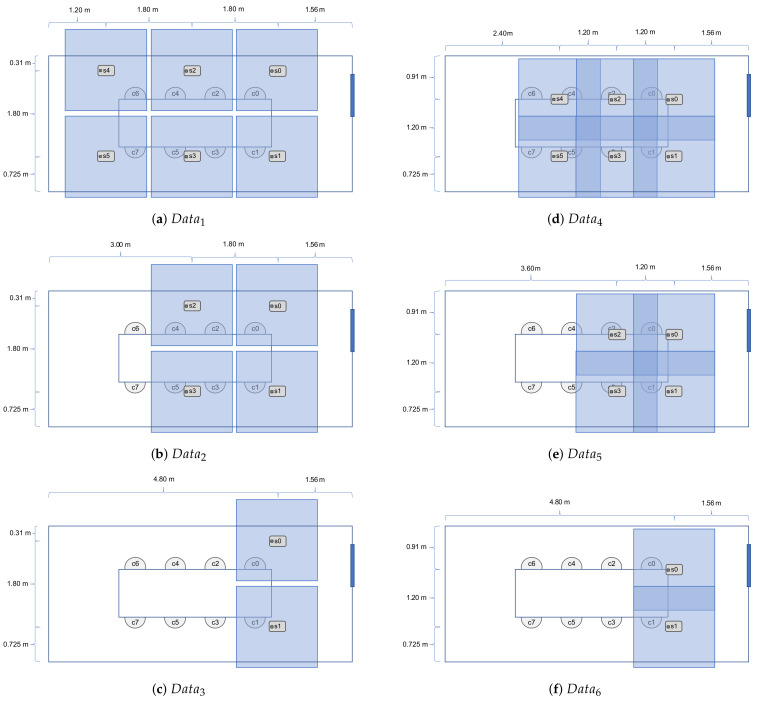
Data collection setups. Each sub-figure shows the setup of the sensors in each of the six different data collections; the corresponding data sets are referred to as $data_{1-6}$. The sensors are shown as grey rectangles. Each sensor’s viewing area is illustrated by an opaque blue square. Subfigures (**a**–**c**) show sensor setup with no overlap, while subfigures (**d**–**f**) show sensor setup with overlap.

We selected a meeting room at our department for multi-sensor data collection (see Figure 1 and Figure 2). We used six low-cost and low-resolution heat sensors (8×8 pixels each) for data acquisition [23]. In Figure 1, we provide a view of the meeting room with chairs and sensors annotated where the experimental data for this study were collected. The photo that is shown in Figure 1 was taken to the rightmost side of the floor plan seen in Figure 2. In our previous work [3], we provided an example of how the viewing area size (at the table level) for one sensor was calculated by using the field of view angle and the ceiling height information. Herein, we used the same simple triangulation technique in order to calculate the view area of each sensor at the table height level:

(1) s=2·(tan(v/2)·h)

where *s* is one side of the viewing area, *v* is the field of view angle, and *h* is the height from the sensor chip to the tabletop surface. Our measurements were h=176.5 cm and $v=60^{\circ }$, resulting in s≈170 cm. These variables can be seen in Figure 3. Figure 2 shows the room empty, while Figure 4 illustrates the sensor setup during the different data collection steps. In Figure 4, the field of view of each sensor is also illustrated with a larger square around the sensor position.

A total of six different data sets was collected; Table 2 shows details of such data sets. $Data_{1-3}$ used a distance of 180 cm between each sensor, whereas $Data_{4-6}$ used a 120 cm distance between each sensor. The total overlap can then be calculated as:

(2) $$o=1-\frac{d}{s}$$

where *o* is the overlap and *d* is the distance between the sensors. Each data set corresponds to a recording over 5 min., where the two participants switched seats every minute (or entered or left the room), resulting in different occupancy values for each time interval (denoted $t_{0-4}$). In Table 2, we have tabulated the details regarding our multi-sensor data set. ID numbers of the active sensors, their field of view overlap percentages, the total number of occupants within the room, and the total number of the data frames from each sensor are presented.

## 5. Methods

**Figure 5:**
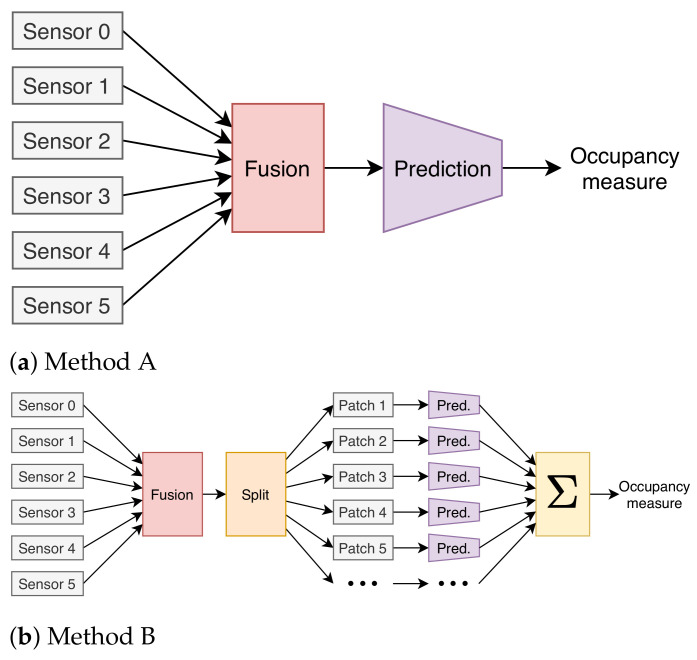
The two different multi-sensor data fusion methods used in our study, Method A and B. (**a**) Multi-sensor information is fused and resized to the network size before applying the prediction algorithm. (**b**) Multi-sensor information is fused first; afterwards, the fused image is split into a certain size of new images where the prediction results are obtained from each of them individually.

In this section, we describe the mathematical steps towards solving the occupancy prediction challenge while using multi-sensor low-resolution thermal images. We provide our approach with a flow chart that is composed of modules in order to make our steps clear and reproducible. In order to solve the challenging multi-sensor data fusion problem, we considered two possible approaches, Method A and B, as illustrated in Figure 5. Those are described hereafter.

### 5.1. Method A

In Method A, the 8×8 pixel readings from each sensor are fused into a single “image” for each time-unit (timestamp) where participants are stationary. Each sensor sends 8×8 pixel readings which are annotated with a timestamp. Each pixel is a temperature reading in the unit of $\frac{1}{10}$${}^{\circ }$C ranging from 0 ${}^{\circ }$C to 80 ${}^{\circ }$C. Because the same timestamps of each sensor are considered when this joint image is created, the sensor synchronization problem has to be solved in this process as well (see details in Section 5.4). The fused image is then resampled to 64 × 64 pixels while using bicubic interpolation to be used as input for our NN-based occupancy prediction module (a CNN-regression network), which returns a single value prediction of the occupancy in the room for each timestamp. We have illustrated the overall method in Figure 5a.

### 5.2. Method B

The second approach, Method B fuses the sensors’ data just as described in Method A but with the difference that, before prediction, the image is split into several smaller patches for individual prediction. This split is custom selected for each of the two sets of data with different amount of overlap in order to split each seat into their own patch. The per-patch prediction is carried out with the same network as in Method A. Method B is meant to emulate prediction of an unspecified number of “images”; when expanding occupancy prediction for larger rooms. This method might be favourable, since more sensors can be added and individual prediction can be applied to each sensor. The reason for using per-seat prediction and not the raw per-sensor prediction is that we do not have pixel segmentation labels of the occupancy for each frame, but we do know which seats were occupied for each timestamp. See Figure 5b for a flowchart of this method.

**Figure 6:**
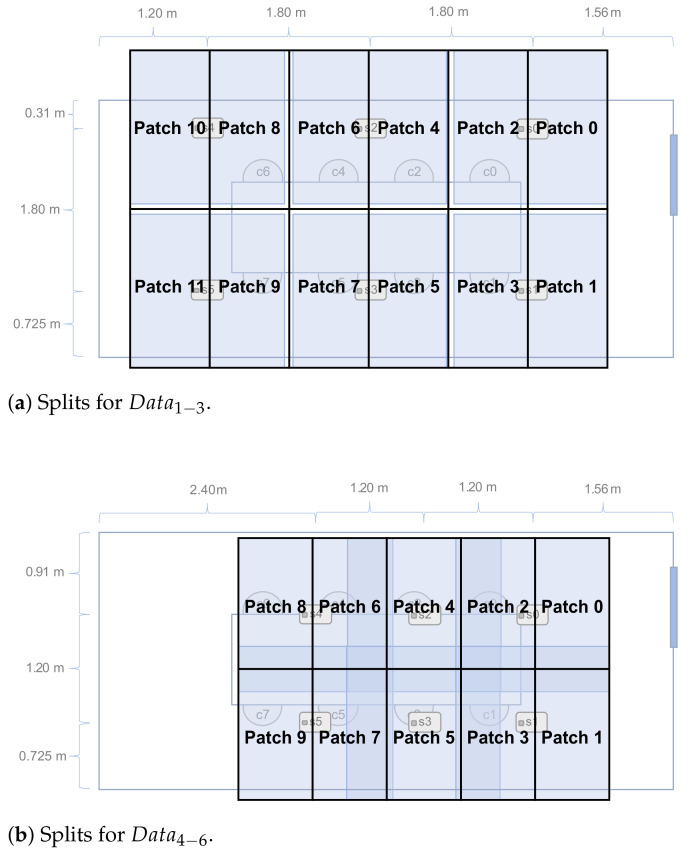
Illustration of how the data sets are split to obtain per-seat patches in Method B.

The splits to obtain per-chair patches are 2×6 for $Data_{1-3}$ and 2×5 for $Data_{4-6}$, see Figure 6 for more details.

### 5.3. Sensor Fusion

In both Method A and Method B, the sensors are first fused. First, the overlay from Table 2 is translated into pixels. 0% is translated as overlay=0 (pixel) and 29.5% is translated as overlay≈2 (pixels). Knowing the number of sensors, their resolution, their placement, and their overlap, the final resulting shape can be calculated using Equation (3).

(3) 8·columns−overlap·(columns−1)×8·rows−overlap·(rows−1)

where columns and rows are calculated, as given in Equations (4) and (5) for our 2×3 set up of the sensors, given the *n* number of sensors listed in Table 2.

(4) $$rows=\left\lceil \frac{n}{2}\right\rceil$$

(5) columns=2

**Figure 7:**
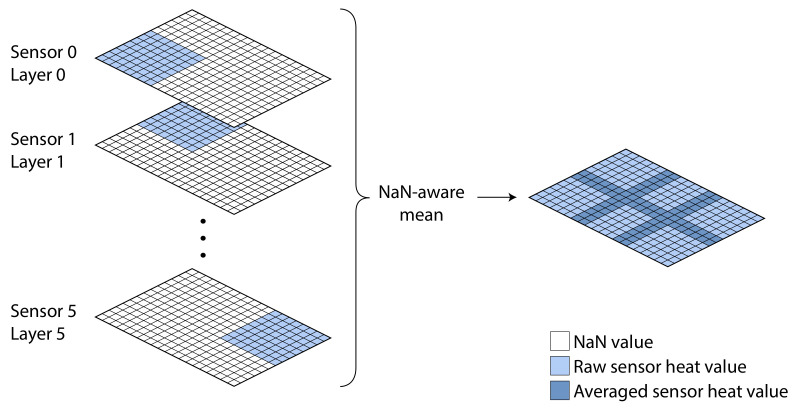
An example of sensor fusion for $Data_{4}$.

Once the final shape is known, the sensors are placed in position in separate layers. These layers are then fused into a single layer, where each overlapping pixel is averaged, while non- overlapping pixels retain their values (see Figure 7). This method requires that the distance between sensors is known beforehand and it has not been tested for missing data in the resulting image.

### 5.4. Sensor Synchronization

Each sensor transmits information roughly once every second. However, this may vary in some cases. In practice, what we observed was that the different sensors used during the same data collection occasion had missing values for one second and multiple values for the next second. Hereafter, we describe how we solved this challenge. First, we made sure that, for each recording, we allowed some seconds before performing the occupant placement. Subsequently, we ensure that all of the sensor data for a certain data collection had the same starting timestamp and ending timestamp. At this point, each sensor’s data had different amounts of entries, even though they should be equal if the sensor would have had a steady rate of one frame per second. We converted all the sensor data to uniform lists for further fusion by stepping through the timestamps, second-by-second, from the start time to the end time, to which the sensor data had previously been limited to. For each timestamp, we checked whether each sensor had any data entry, and picked those values. If any sensor did not have any entry, we checked whether the previous timestamp yielded any entry for the given sensor, and repeated until found. If we did need to check previous timestamps and a timestamp was found to contain multiple entries, then the last of those entries was chosen (the closest one to the second we were looking for). If a timestamp was found for the current timestamp we were looking for (not looking backwards), the first entry was always selected.

### 5.5. Predicting Occupancy Using a CNN Regression Network

We built a predictive model employing a Convolutional Neural Network (CNN) that has as a goal to give a single output, i.e., how many people there are in the observed input. Because we trained a regression network, we set the optimization criteria as the Mean Square Error (MSE/L2-norm) between the target and the output.

The network consisted of seven down-convolutions with a leaky rectified linear unit (ReLU) of slope −0.2 followed by 3 fully connected linear layers that predict a single value with a normal rectified linear unit at the very end. Leaky ReLU is known as the most popular extension of the ReLU activation function, which has frequently been used in the literature. Leaky ReLU relaxes the classical ReLU function by also allowing small negative values to contribute to the results. Even though using a leaky ReLU activation function instead of the classical ReLU does not make a significant impact on the optimization performance, earlier research has shown that, while using leaky ReLU NNs, the convergence is slightly faster, which is perhaps due to the difference in gradient among the two rectifiers [24]. No normalization layers were employed in the network, as they did not grant any increased performance during early experimentation.

The network used an input of 64×64×1, which means that all of the inputs need to be resized to this resolution; this was done by applying bicubic resampling. This can be a disadvantage for Method A, since the network has to learn to classify for 6 quite different looking shapes, even though they are resized, whereas Method B has a more uniform size of resampled images.

Method A is trained to detect 0–2 people per input data, while Method B is only trained to detect 0–1 people, one per seat. There is also the special case for $t_{2}$, where one person is standing between sensor 0 and sensor 1, this person is counted as 0.5 for patch 0 and patch 1.

For both of the methods, all the temperatures are standardized while using the per-pixel mean and standard deviation of the entire dataset during both training and evaluation. During training, augmentation was also employed: random rotation, flipping, and shearing.

### 5.6. Training the CNN Regression Network for Occupancy Prediction

For each occupancy time *t*_0–4_, there is a label with how many people there is in the whole frame. Each time *t* has a label for the whole collection of sensors. That is, if there is a subject at sensor *s_0_* and nowhere else, the whole combined frame for the data sequence gets the same label. Each time that *t* also has a transit period where there is no label, in this period the test subjects are switching seats. Therefore, these data are removed, either due to it being hard to judge where and when a person is visible, or that the test subjects are in transit, so they can go out of view from the sensors.

Each time that *t* is roughly 60 s. When subtracting the transition frames, we have around 55 frames ± 3 frames. The first three minutes ($t_{0-2}$) are used for training the CNN model and the last two minutes ($t_{3-4}$) are used for evaluation. During training, 60% of the data is further divided into training (85%) and validation (15%). During training, the data are retrieved in random order with the model having no temporal knowledge between the training frames. After each training session, the model is tested on the validation data in order to see the progress made between epochs. We train the model for 1000 epochs, and then select the weights for the model that performed the best on the validation set. When training is complete, we evaluate our model with the evaluation set using the measures mean squared error, mean absolute error, accuracy, and confusion matrices.

### 5.7. Model Training Settings

**Figure 8:**
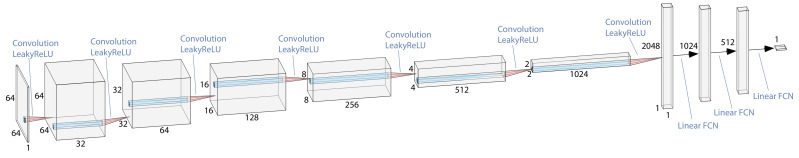
The Convolutional Neural Network (CNN) regression network architecture. It consists of seven convolution layers, followed by three fully connected layers.

When training the model, we employed the commonly used AdamW optimizer. The following settings where applied when training the model; test percentage = 0.15 (amount of data used for testing during training), batch size = 100, learning rate = 0.00001, weight decay = 0.00001, beta1 = 0.9, beta2 = 0.999, epochs = 1000. The model shape can be seen in Figure 8. The leaky ReLU layers had a negative slope of 0.2.

## 6. Experimental Results

Our model is lightweight, which implies a fast training and inference speed. This is due to our input data being of low dimensionality and low resolution. We also chose a limited amount of convolutional and linear layers to decrease the model complexity. The training and inference were evaluated on both an Nvidia GTX 1070 and an Nvidia RTX 3090. On both graphics cards, the training phase (of 1000 epochs) for Method A was around 30 min., while the training phase of Method B was around 97 min. On both graphics cards for both methods, inference took around 2 ms at most. Predicting multiple sensors in parallel was only limited to available graphics memory and it did not increase inference time. The low inference time means that 500 sensors could even be predicted in sequence and still be considered as real-time, given the sensor capture rate of one frame per second.

Inference was also examined on two CPUs, AMD Ryzen R7-2700X and an Intel Core i9-9900K. For both CPUs, inference took around 50 milliseconds at most. With previous calculations in mind, 20 sensor batches could be processed in parallel per second, where the batch number is dependent on the number of CPU threads. Whether a GPU or a CPU was used for inference, we could still perform prediction in real-time with our given set up.

**Figure 9:**
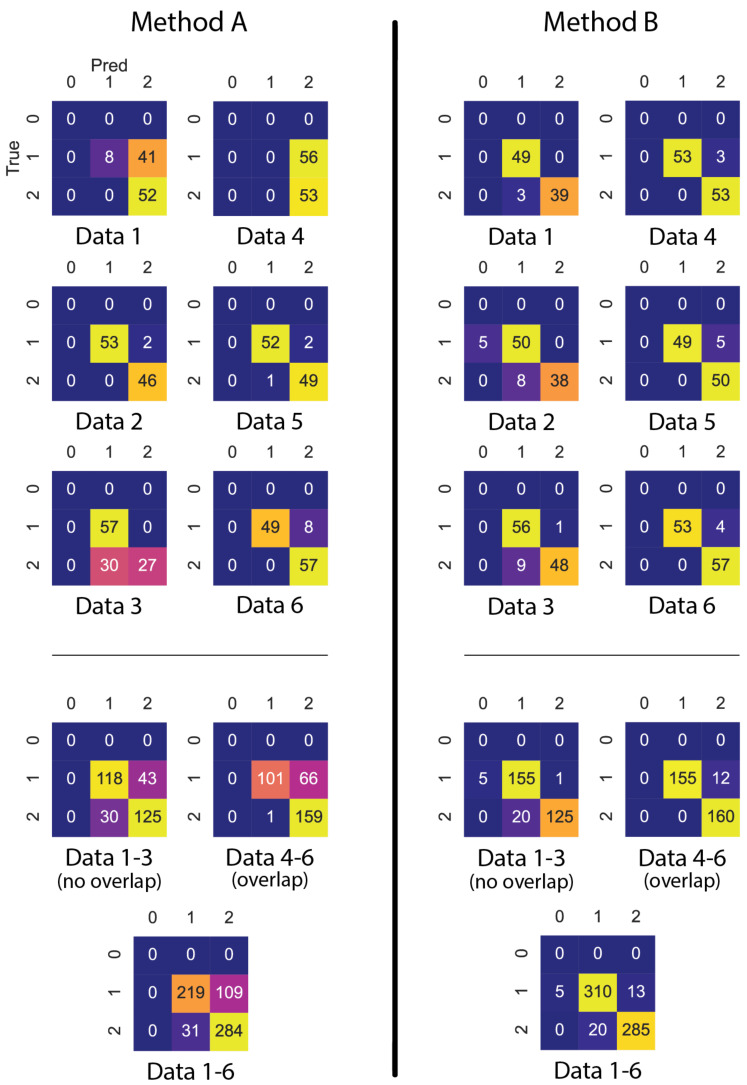
Occupancy prediction confusion matrices for method A and method B. The top six confusion matrices represent validation results for try 1–6 for both methods. The lower three represent the cumulative confusion matrices for the aforementioned confusion matrices. At each confusion matrix, horizontal and vertical 0, 1, and 2 numbers indicate the number of the predicted and the real occupant numbers, respectively.

We compare the CNN-based method’s detection results with a manually labelled multi-sensor evaluation set ($t_{3-4}$) to assess the performance of our methods. The confusion matrices that are shown in Figure 9 provide the performance of our solution based on the number of occupants predictions at six different attempts. In such a figure, the performances for True Positive, False Positive, and False Negative predictions can be seen for each predicted class (0 people, one people, two people). Along the diagonal (starting at the top-left corner) is the True Positives for each class, along the rows we can obtain the False Negatives for each class (excluding the True Positives) and, along the columns, we can get the False Positives for each class (excluding the True Positives). Note that the usage of the classes 0, 1, and 2 people present in the confusion matrices is due to those being the only three classes that were predicted by our model. The target classes were only one or two people present, but for one data ($data_{2}$ using Method B), 0 people present was sometimes inaccurately predicted. The model is capable of predicting even more "classes" (i.e., 3, 4, 5, ..., *∞*), but the confusion matrices are limited to just the classes that are predicted for the given evaluation data.

In these confusion matrices, we notice one significant anomaly in the results of $Data_{1}$ and $Data_{4}$ when they are processed with the fusion Method A. During the data collection process of $Data_{1}$ and $Data_{4}$, the computer that captured the multi-sensor data was standing on the table below sensor 5. This resulted in a small hot-spot that might have affected the results of Method A. Interestingly, this does not seem to be the case for Method B. This could be explained by the different sensor data fusion and different training scheme employed for the Method B compared to the Method A. This anomaly appears to be significant for the rest of the data for Method A. Method B does not have such large deviations in the predicted results, which shows that it might be a more robust approach when comparing both methods.

### 6.1. Comparing the NN-Based Method with Earlier Published Methods

In the following subsections, we describe the comparison of our new NN-based occupancy prediction algorithm with the machine learning (ML) and computer vision (CV)-based methods proposed in our previous study [3]. As we mentioned earlier, these previously suggested two methods were designed for single sensor data processing. Therefore, in order to be able to process multi-sensor data with these solutions, we used two sensor fusion methods, Method A and B, as illustrated in Figure 5. We have replaced the NN-based prediction module of Method A and Method B with the CV and ML-based prediction modules, respectively. However, we did not achieve reliable occupancy prediction results and we concluded that both of the fusion methods do not seem to be a good fit for the earlier published solutions. In the following subsections, we provide more details of the comparisons.

#### 6.1.1. Comparison with the Computer Vision–Based Method

The previously proposed CV-based method is designed to work when an empty room recording of a heat sensor is available. This empty room recording is used for removing the noise artifacts that might be coming from other warm objects in the environment (like a radiator, computer, screen, a hot drink, or a surface heated by direct sunlight).

In the multi-sensor data set (Table 2), an empty room recording was only available at the $Data_{6}$ recording attempt during the time period represented with the $t_{0}$ label. Hence, we used this specific time period data for the background compensation step of the CV-based method and we used the whole $Data_{6}$ time stamps for testing the CV-based method. First, we selected the sensor data fusion Method A, replacing the NN-based prediction module with the CV solution. Unfortunately, we did not obtain a high occupancy prediction performance, as we did in the single sensor experiments published. When we made the comparison, we calculated a RMSE value of 1.85. This means that, at each time stamp, the predictions provided approximately two persons extra or less than the real value. We believe that the main cause of this high error and low performance of the multi-sensor based CV system comes from the background compensation step of the algorithm. The purpose of the background compensation step is to correct each pixel value if there is any noise coming from other heated objects in the room. However, after applying the fusion method, as described for Method A, the fused image pixels do not contain the original background value (because of the image resizing operation) and, therefore, the background noise at any specific point cannot be compensated.

When we used the CV-based prediction method within Method B (by replacing the NN-based prediction module with the CV-based solution), we, unfortunately, obtained even much higher errors than with Method A, showing unreliable results. First, since the sensors in $Data_{6}$ have overlapping field of view, the overlapping pixels are fused by calculating the mean of the pixel values. In this way, the original pixel values of each frame and also the background calculation frames are lost. Thus, the background compensation method cannot counterbalance the noise effects, since it does not know the original pixel values and the noise effects. Secondly, if a person is sitting between two sensor views, then the slight impression of the person in each sensor is not sufficient heat to predict occupancy (creating a maximum of one highlighted pixel in each image patch after the splitting process).

#### 6.1.2. Comparison with the Machine Learning-Based Method

Finally, we have compared the new NN-based method with the previously proposed ML-based solution. To do so, once again, we considered both Method A and Method B as fusion techniques. Because, in our previous work, we have already trained a ML model that can make occupancy predictions, we have simply replaced this trained module with the NN-based prediction module. In Method A, we noticed that the deformed (fused and resized) input image does not provide similar feature characteristics to the single sensor data that we have used for training the ML based model. Thus, the algorithm was not able to make any meaningful predictions. The ML based model needed to be re-trained by creating a big data set. We have avoided this highly time consuming process since we also did not expect this solution to provide more accurate results than the NN-based solution which has learned which kind of features to look for in fused images. Secondly, we considered Method B and tried our pre-trained ML module instead of the NN-based prediction module. Again, we noticed that the ML model needed to be re-trained in order to learn the situations when a person sits between two sensors for different field of view overlap ratios. For similar reasons as with Method A, we have decided to rely on the NN-based prediction module that has already learned such special situations.

## 7. Discussion

In the following sub-sections, we discuss the advantages, applicability, and limitations of the proposed solutions. We also address the influence of sensor field of view overlap on the performances.

### 7.1. Comparing the NN-Based Methods with the Traditional CV- and ML-Based Methods

There are various CV- and ML-based occupancy prediction techniques for processing low-resolution heat sensor data in the literature. In order to compare the solutions that are presented in this paper with these types of methods, we used our earlier CV- and ML-based solutions presented in [3] with the multi-sensor data sets used in this study. To do so, we replaced our previous CV- and ML-based prediction modules with the NN-based prediction module in Method A and Method B. We list our observations from this comparison, as follows:

- When the CV-based method is used with Method A, because of the lost original pixel values during the fusion process, the method could not compensate the background noise artifacts coming from each individual pixel. Therefore, the CV-based method provided inaccurate predictions after the unsuccessful background noise compensation step.
- When the CV-based method is used with Method B, again the method could not successfully compensate the background noise because of the lost original pixel values for the overlapping pixels. Once again, the CV-based method provided inaccurate predictions due to the unsuccessful background noise compensation.
- When the CV-based method is used with Method B, even when the sensors’ field of views do not overlap, the occupancy predictions could not be made correctly when the occupants appeared between two sensor field of views.
- When the ML-based occupancy prediction method is used with Method A, the previously trained occupancy prediction model could not make any successful occupancy prediction, since the fused image did not provide features which are similar to the features which were extracted from single sensor data during the training process.
- When the ML-based occupancy prediction method is used with Method B, we observed that, for different sensor field of view overlaps and for different occupant sitting positions (appearing between two sensor field of view or not), the ML-based method needed to be re-trained to learn the features of such specific cases.

In summary, we have not achieved a reliable solution by simply replacing the previously developed CV-based or the ML-based prediction modules with the NN-based prediction solution that is presented in this paper. We do not want to imply that these two traditional solutions are obsolete when they are compared to a NN-based solution. As we have shown in our earlier article with extensive experiments [3], these two methods present good performances (that are comparable to the NN-based method) while using data that were collected with a single heat sensor. However, these two methods come with their respective advantages and disadvantages, as we have discussed in detail in [3]. We believe that these traditional methods would require fewer data to be trained as compared to the NN-based method. However, the proposed multi-sensor fusion methods do not seem to be suitable to be used with the traditional techniques by simply changing the prediction module.

We see that the main reason for having high performance from the proposed NN is that it learns how to assess the fused sensor information, according to the comparison made with the previous CV-based and the ML-based methods. The multi-sensor fusion process of the Method A and the fusion followed by the split module in Method B distort the real values of the original sensor pixel information, as we have explained in Section 5.3. The CV-based method relies on the spatial accuracy of the input image and the real heat values of each pixel, therefore, it cannot count occupants in the distorted fusion images. The ML-based method similarly relies on the heat related features extracted from the raw sensor images, therefore, again, this method cannot find meaningful features to label the scene with the number of occupants. Nevertheless, the proposed NN can learn how to extract meaningful information from a distorted multi-sensor data; therefore, it works reliably.

### 7.2. Choosing a Reliable Method to Observe Occupancy with Multi-Sensor Data

We have proposed two different fusion methods, as illustrated in Figure 5. Whereas they differ in the prediction phase, they share the same initial fusion step, where overlapping areas are averaged from a manually measured distance. We deem this initial fusion step necessary for any kind of multi-sensor set up, at least when the sensor areas overlap. This is because it is necessary not to count a person in the overlap area twice.

From the results, we speculate that Method B is not only the most reliable method when it comes to performance, it is also, in theory, more generalizable for irregular sensor setups and for covering much larger office spaces. Because Method A needs to look at the entire fusion “image” of all sensors, it needs a full N×M array of sensors (in this experiment 2×3, 2×2, 2×1) and it quickly becomes unfeasible when adding further sensors.

Method B has the benefit of performing per-sensor predictions where the sensor set up can be completely irregular. It should be noted that we have only tested Method B in a very basic setting, where, instead of predicting per sensor (and having multiple classes), we have predicted smaller patches of only 0 or 1 people present (and 0.5/1.5 in a very special case).

While we believe that Method B is superior, it is not clear that is the case for all possible set ups. For example, Method A outperformed Method B for the four sensors set up ($data_{2}$ and $data_{5}$), even if only barely. This might suggest that Method A is more suitable for the very specific case of an N×N sensor array set up.

### 7.3. Influence of the Sensors Field of View Overlap on the Prediction Performance

Our experimental results show that having a higher overlap in a small area increases the precision of the occupancy prediction results; however, higher overlap creates a negative impact when a larger area is covered with more sensors. This might be good news when the sensor costs for covering large areas are concerned. Our experiments show that it is possible to install the sensors sparsely and observe the occupancy with high precision accuracy.

## 8. Future Work

### 8.1. Robustness to the Environmental Changes

In our study, the data collection environment did not have any windows and it was artificially illuminated. This is quite common in business buildings. Therefore, in this study, we have not analyzed the effect of weather and illumination changes on the performances of our algorithms, as we did in our earlier study [3]. In such earlier work, we employed eXplainable AI (XAI) methods to interpret the performance of the feature extraction methods in an ML-based algorithm in order to predict occupancy when the sun illumination changed within a 24 h recording in a smart office room with large outside windows. Therefore, we are planning to carry out a similar analysis with the multi-sensor setting and the current CNN solution in the future.

### 8.2. Prediction Models Considering the Occupancy Dynamics

A few occupancy dynamics observational studies have been presented in the literature. In the future, we are planning to apply the algorithms presented here to even larger areas, such as large business spaces or a university building with many offices, meeting rooms, and classrooms. In such cases, we believe that we will be looking for more predictive models to estimate the future occupancy states of each room when the activity on other building areas is known.

### 8.3. Optimized Training for CNNs

For configurations, such as in Method B, the network could be trained using multiple objectives. In this study, we trained the network on each individual seat patch and later summarized the predictions to a single value. While the network was trained only to predict each seat patch, they could also be trained to accurately predict a summarized value. An objective like this (perhaps with additional information about the position of the sensors) could possibly solve the challenge of sensor overlap in an unsupervised manner.

In this study, we use a single network architecture for both Method A and B. It could be worth exploring the use of smaller networks that do not require a 64×64 input. While bicubic interpolation could be useful for the network, up-scaling might only be needed to a resolution of 16×16 if at all. This would make the inference speed even faster, which makes the network viable for edge devices.

### 8.4. Optimal Sensor Placement

Another important open research topic is to find a reliable and practical algorithm in order to solve the optimal sensor placement problem. The position and number of sensors are vital for building occupancy estimation. In the literature, only a few works have been carried out to investigate the optimal sensor placement for the task of building occupancy estimation and detection. A systematic and theoretic analysis of optimal sensor placement is an urgent and challenging task, where further research is required. In light of our experimental results regarding the impact of the field of view overlaps between different sensors, in the future, we would like to investigate this challenge and propose optimal sensor placement solutions. We would like to test our algorithms in buildings that have more complex floor plan geometries and buildings that have restrictions regarding sensor placements.

### 8.5. More Extensive Data Collection

The data used in this study were limited to 0–2 people visible at any time and only using regular N×M sensor setups. The result was that our evaluation dataset, while being aimed for regression, only contained two different target classes: one or two people present (see $t_{3}$, $t_{4}$ in Table 2). Note that the training dataset contained more classes, and both of the methods are capable of predicting any non-negative real number (which is often rounded to an integer). Having the opportunity to record better data, for larger rooms, with more participants could give further insight into the viability of our proposed methods. This is especially true for Method B, where a larger and better annotated dataset could make sure that the method generalizes better.

### 8.6. Data Cleaning and Compression

Finally, we would like to address several issues that are highly common when real-life data and applications are considered. One of these issues regards missing data due to a broken sensor or due to a synchronization problem that might happen to one or more sensors. We need to modify our workflow in order to deal with such a challenge. First, we would need to add algorithms that could determine whether the sensors are working properly and are sending synchronized data or not. Secondly, we would need to re-design the fusion and prediction modules in order to provide reliable results.

Another issue regards the increasing amount of data when the data acquisition is carried out in large buildings 24 h a day. In order to deal with the associated challenges of large amounts of data, we would need to, for example, look for suitable data cleaning (removing data that are not useful) and data size reduction methods.

## 9. Conclusions

In this paper, we have proposed two different multi-sensor fusion methods and an NN-based prediction solution, in particular, a CNN, in order to determine the occupancy of large indoor environments using low-resolution and low-cost heat sensors. We have discussed the advantages and disadvantages of different sensor data fusion methods when considering their impact on the prediction performances and their extendibility possibilities.

The experimental results of our solutions show high-performance accuracy and real-time prediction capabilities. We also analyze the impact of field view overlaps, undertaking experiments to observe the prediction performance when the field of view overlap spams from zero to higher values. We believe that our suggested solutions, the experimental designs, and obtained results contribute to finding occupancy prediction solutions for future smart spaces.

One of the major limitations of the proposed study is that it needs modifications if it was to be used in offices with complex floor plans (such as L-shaped, U-shaped, triangle, or other geometries that are not square or rectangle). The proposed sensor fusion algorithms are capable of dealing with square or rectangle office floor plans when the sensors are installed in a rectangular grid structure. Thus, in our future work, we would like to study these two related challenges: (1) developing sensor placement optimization algorithms to suggest the best sensor positions to cover any office floor plan and (2) improving the sensor fusion methods in order to be able to predict occupancy for complex office floor plans that might also imply complex sensor placement distributions. Finally, two possible extensions of this work are to consider more crowded spaces and different environmental conditions that affect the data, such as exposure to variations of daylight through windows.

## Figures and Tables

**Table 1:** The most frequently used occupancy prediction sensors are represented with their advantages and disadvantages when smart office applications are concerned.

Sensor	Measure	Capabilities	Limitations
PIR [6]	It measures infrared light radiating from objects in its field of view.	It acts as a motion detector, therefore it might be effective for knowing how much activity occurs in a certain area.	The sensor doesn’t enable knowing the number of occupants. Even dust or flies might cause false activity detection. Multi-sensor placement, multi-sensor data fusion and large area coverage problems are not solved.
Smart meter [7,8]	It measures the energy consumption.	A single sensor might help to estimate the number of occupants within a whole building.	Energy usage and the occupancy correlation of each building highly depends on the activities within the building, therefore how to generalize a method developed for one building to other buildings is not known.
CO${}_{2}$ sensor [9]	It measures CO${}_{2}$ levels.	Only one sensor might help to estimate the total number of occupants within a room.	The sensor data will not be reliable when the doors/windows are open or when a ventilation system is working.
Heat sensor [3]	It works like a very low-resolution heat camera.	One sensor located on the ceiling can see approximately 2.5 m${}^{2}$ area. It might provide a good estimation of the number of occupants within the field of view.	Optimal sensor placement, multi-sensor data fusion and large area coverage problems are not solved. (We focus on potential solutions in this study.)

**Table 2:** The multi-sensor data set acquired with six sensors as represented in Figure 1. The “Sensors” and the “Occupancy” columns indicate the sensor IDs that were active and the total number of occupancy within the room. “Total frames” column shows the number of frames that were collected from each different sensor at six different data acquisition attempts.

Data ID	Sensors	Overlap (%)	Occupancy					Total Frames
$t_{0}$	$t_{1}$	$t_{2}$	$t_{3}$	$t_{4}$
Data${}_{1}$		0	1	2	2	2	1	301
Data${}_{2}$	0, 1, 2, 3	0	1	2	2	2	1	300
Data${}_{3}$	0, 1	0	0	1	2 ${}^{a}$	2	1	301
Data${}_{4}$	0, 1, 2, 3, 4, 5	29.5	1	2	2	2	1	303
Data${}_{5}$	0, 1, 2, 3	29.5	1	2	2	2	1	305
Data${}_{6}$	0, 1	29.5	0	1	2 ${}^{a}$	2	1	301

^a^ One person is standing.

## Data Availability

Experimental data set can be found at https://sites.google.com/view/occupancy-prediction/home.

## Abbreviations

The following abbreviations are used in this manuscript:

AI	Artificial Intelligence
CNN	Convolutional Neural Network
FN	False Negative
FP	False Positive
HVAC	Heating, Ventilation, and Air Conditioning
IoT	Internet of Things
ML	Machine Learning
NN	Neural Network
PIR	Passive Infrared
ReLU	Rectified linear activation function
TP	True Positive
XAI	eXplainable AI
